# Nutritional analysis of vegan recipes: From social media to plate

**DOI:** 10.1002/fsn3.4382

**Published:** 2024-08-02

**Authors:** Tuba Yoldaş, Gözde Sultan Kaya, Ayhan Parmaksız, Handan Işıklar, Elif Günalan

**Affiliations:** ^1^ Department of Nutrition and Dietetics, Faculty of Health Science Istanbul Health and Technology University Istanbul Turkey; ^2^ Department of Biostatistics, Faculty of Medicine Istanbul Health and Technology University Istanbul Turkey

**Keywords:** recipe, vegan, vegetarian, vitamins

## Abstract

The study aimed to determine the nutritional composition of vegan recipes shared on the Instagram social media platform and to compare these ingredients with their non‐vegan/omnivorous versions. Turkish and English vegan recipes of meatballs (*n* = 53), burgers (*n* = 12), pizza (*n* = 15), pasta (*n* = 38), cake (*n* = 58), and cookies (*n* = 52) were obtained from Instagram. They all met the criteria of being shared as #vegan labeled, presented detailed information about ingredients, and only included plant‐based ingredients. Afterward, non‐vegan/omnivorous recipes (*n* = 228) were determined as equivalent to the vegan recipes in each food group, and a total of 456 recipes were evaluated. The amounts of macro‐ and micronutrients per 100‐gram serving were obtained by the Nutritional Data System (BeBiS). According to the outcomes, the most significant differences in nutritional composition were found between vegan and non‐vegan meatball recipes. The levels of cholesterol and B12 were significantly lower in English vegan recipes of meatballs, pasta, cake, and cookies compared to their non‐vegan versions (*p* < .05). Consuming different vegan foods throughout the day could provide complementary nutrient intake and sustainable optimal health. Nevertheless, recipe‐based updates could be an innovative approach in future vegan studies. In addition, analysis of vegan recipes could be considered to modulate vegan supplementation programs.

## Graphical abstract

This study explores the nutrient composition of vegan recipes on Instagram and compares them with their non‐vegan/omnivorous versions. Vegan meatball and cookie recipes have lower cholesterol and vitamin B12 levels when compared to non‐vegan recipes. In addition, fiber levels are significantly higher in vegan meatball and pasta recipes. Consuming different foods throughout the day can sustain optimal health thanks to complementary nutrient intake in a well‐planned vegan diet.

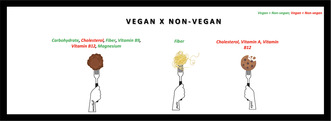

## 1 INTRODUCTION

Vegans exclude eating all foods of animal origin, while vegetarians avoid foods that involve killing animals (Larpin et al., 2019). A vegan diet, which is also considered a plant‐based diet (Storz, 2022), includes all minimally processed fruits, vegetables, whole grains, legumes, nuts and seeds, herbs, and spices, and excludes all animal products, including red meat, poultry, fish, eggs, and dairy products (Ostfeld, 2017). The positive effects of plant‐based nutrition on health, religious beliefs, and ethical and ecological views motivate individuals to adopt a diet with such strict rules (Pilař et al., 2021). In addition, technology for information transfer on the internet and social life triggers the spread of veganism with an increase in population size and education level (Erden & Balaban, 2017). The total proportion of vegetarians and vegans in Türkiye is below 5 percent of the population (*n*≈80,000) (Guler & Caglayan, 2021). The rapid spread of veganism in the population and the fact that the vegan product and service sector is a growing economy requires the investigation of this consumption pattern and nutrition culture (Mercan et al., 2017). Vegan nutrition‐related studies in the scientific literature are mostly related to the problems vegans encounter in restaurants and social life, the effects of vegan nutrition on general health, and nutritional deficiencies (Craig, 2009; Dwyer, 1991; Key et al., 2006; Mercan et al., 2017).

The limited intake of saturated fat and cholesterol in vegan diets has some positive effects on health. In this context, serum total cholesterol, HDL cholesterol, LDL cholesterol, glucose, body mass index, and blood pressure are lower in vegan individuals than omnivores (Craig, 2009; Dinu et al., 2017; Hovinen et al., 2021). Also, vegans have less risk of developing coronary artery disease and cancer (Dinu et al., 2017; Shah et al., 2018). On the other hand, the literature indicates that removing all animal‐source foods from the diet can cause malnutrition‐related outcomes. In particular, iron (Chouraqui et al., 2023), vitamin D (Schüpbach et al., 2017), vitamin B12 (Alexy et al., 2021; Selinger et al., 2019), calcium (Alexy et al., 2021; Sarıkaya et al., 2023; Schüpbach et al., 2017), zinc (Sarıkaya et al., 2023), alpha‐linolenic acid (ALA) (Welch et al., 2010), eicosapentaenoic acid (EPA), and docosahexaenoic acid (DHA) (Neufingerl & Eilander, 2021; Welch et al., 2010) intake is lower in vegan individuals than in omnivores. The chronicity of these nutritional deficiencies can lead to a wide scale of health problems. Despite all the contradictions, the number of individuals adopting a vegan diet has increased rapidly. In this context, the widespread use of social media in nutrition, as in every field, can be a triggering factor (Chung et al., 2017). In recent studies, social network analysis about vegans on social media platforms has drawn attention. In this context, the social network and content analysis of Yegen and Aydın (2018) have emphasized animal rights and equality between species (Yegen & Aydın, 2018). Another nutrition‐related social network analysis study reported that vegan, homemade, and organic foods posted on Twitter are perceived as the healthiest (Pilař et al., 2021). According to a social network analysis of plant‐based diets and veganism, Twitter posts about veganism contain more recipes, trends, criticisms, and negative thoughts about veganism (Aleixo et al., 2021). In this context, research on social media and vegan nutrition generally covers mood analyses of individuals. However, social restrictions are not the only problem for vegan individuals. In vegans, poorly planned recipes and barriers to a vegan diet are also significant problems (Alcorta et al., 2021).

There is an analysis of the nutritional content of clean eating trend recipes in various blogs (Dickinson et al., 2018). However, no nutrient analysis studies present about vegan recipes. This study aims to determine the nutritional composition of Turkish and English vegan recipes shared on the Instagram social media platform and to compare these contents with their non‐vegan/omnivorous forms. With this approach, food‐specific information about the nutrient profiles in the mostly searched and shared vegan recipes on Instagram can be obtained.

## 2 METODOLOGY

### 2.1 Study design

This descriptive research focused on the nutritional evaluation of online vegan recipes, and no survey or questionnaire was applied within the scope of the study. According to Google Trends (GT) data, internet‐based searches for vegan nutrition increased worldwide. GT was a beneficial, freely accessible tool for evaluating various aspects of web‐based human behavior (Mavragani et al., 2018). For this reason, GT data was considered when designing the study. According to GT, searching for vegan foods was mainly about finding restaurants, nearby foods, and recipes (Google Trends, 2024).

The food groups in the study were selected based on the most searched and shared recipes. Firstly, vegan versions of soups, stews, casseroles, cheese, yogurt, meatballs, burgers, pizza, pasta, cake, and cookies were searched on GT. The most search ranking for vegan foods was vegan cake, vegan cheese, vegan pizza, vegan burger, vegan pasta, vegan cookie, vegan casserole, vegan meatballs, vegan salads, vegan soups, vegan yogurt, and vegan stews. In the cheese category, it was not possible to determine the nutritional content accurately and reliably due to water loss and volume change during cooking. Therefore, vegan cheese was excluded from the study. In addition, the four least searched foods (vegan salads, vegan soups, vegan yogurt, and vegan stews) were also excluded from the study. The search rankings of vegan casserole and meatballs were close (Google Trends, 2024).

The study was conducted on vegan recipes shared on the Instagram social media platform. Instagram started in 2010 as a photo‐sharing network, with users posting photos edited with digital filters. Then, it extended to a video‐sharing platform (Tingley et al., 2022). It became one of the world's most‐used social media platforms, and the number of Instagram users was around 1 billion (Statista, 2021a). In Türkiye, there were 49,830,000 Instagram users (Statista, 2021b).

**FIGURE 1:**
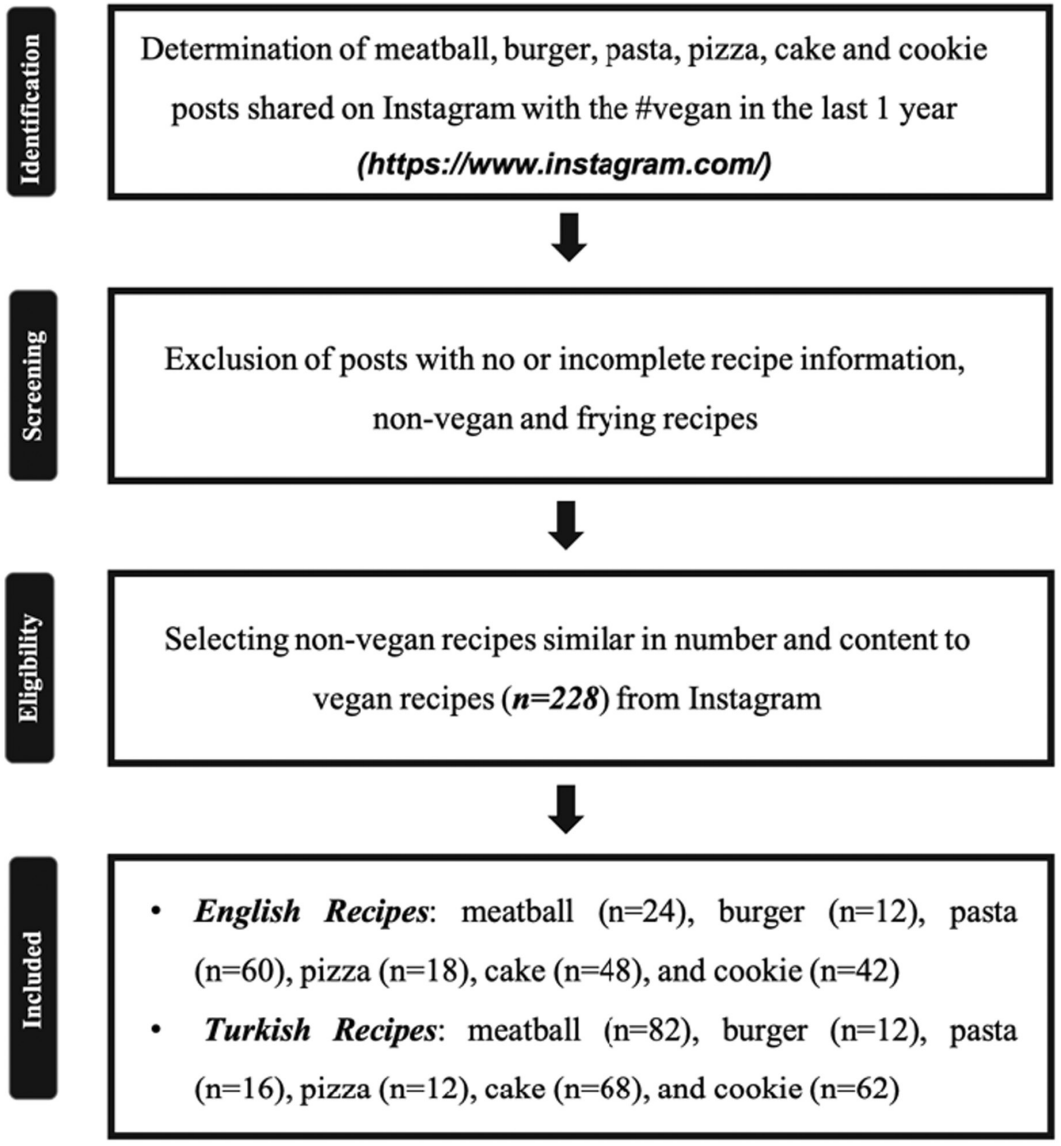
Workflow of recipe selection.

Secondly, the most shared vegan foods on Instagram were determined with post numbers. Accordingly, the total share numbers of foods on Instagram were #vegancake (*n* = 1,200,000), #veganpizza (*n* = 81,500), #vegancookie (*n* = 71,100), #veganburger (*n* = 70,700), #veganmeatballs (*n* = 46,100), #veganpasta (*n* = 31,900), and #vegancasserole (*n* = 7701). Among these items, vegan casserole was excluded from the study due to the lower post number. Ultimately, this study included vegan and non‐vegan meatballs, burgers, pizza, pasta, cake, and cookie recipes shared in Turkish and English (Figure 1). All of the non‐vegan recipes were omnivorous.

### 2.2 Data collection

The recipes in the study were required to comply with the following criteria (Dickinson et al., 2018):

- Recipes must be shared within 1 year (1).
- Recipe contents and quantities must be given clearly, as well as raw and non‐frying (2).
- Both vegan and non‐vegan recipes must have similar ingredients except that animal sources foods in non‐vegan recipes (3).
- Recipes must be standardized in Turkish and English (4).

Foods that did not meet these criteria were excluded from the study (Figure S1).

This study was conducted on Turkish and English recipes. Accordingly, the numbers of included Turkish vegan recipes were meatball (*n* = 41), burger (*n* = 6), pizza (n = 6), pasta (*n* = 8), cake (*n* = 34), and cookie (*n* = 31). On the other hand, the numbers of English vegan recipes were meatball (*n* = 12), burger (*n* = 6), pizza (*n* = 9), pasta (*n* = 30), cake (*n* = 24), and cookie (*n* = 21). Then, non‐vegan recipes (*n* = 228) were selected as equivalent to the vegan versions for each food group. The project was carried out in two stages. In order to standardize, familiarize, and establish the method, Turkish recipes were first researched. According to that, the time frame for Turkish recipes was from 1 August 2021 to 31 July 2022 (1st stage), while the timescale for English recipes was from January 30, 2022 to January 31, 2023 (2nd stage).

The amounts of macro‐ and micronutrients per 100‐gram serving of all recipes (*n* = 456) were calculated by the Nutritional Data System (BeBiS). BeBiS software program can be utilized in medical nutrition therapy, menu planning, food analysis, research, education, and various application areas (*Nutrition Information System‐ BeBiS, Version 8.2, 2019*). It has more than 130 nutrient analyses of more than 20,000 foods. It can be reported that nutritional elements such as calories, protein, carbohydrates, fat, vitamins, minerals, amino acids, fatty acids, oxygen radical absorbance capacity (ORAC), and glycemic index contents of foods depend on their portion and entered amount. The present study preferred menu planning and recipe analysis as the BeBIS functional area and ingredients were entered as raw according to recipe standardization criteria.

### 2.3 Statistical analysis

All measurements were given with median and quartiles. Energy, carbohydrate, total fat, cholesterol, protein, dietary fiber, vitamin A, vitamin D, vitamin E, vitamin K, vitamin B1, vitamin B2, vitamin B3, vitamin B6, vitamin B9, vitamin B12, vitamin C, sodium, potassium, calcium, magnesium, phosphorus, iron, zinc, ORAC, omega‐3, omega‐6, and glycemic index levels were compared between vegan and non‐vegan recipes. In this context, independent two‐group comparisons were performed using the Mann–Whitney U test. In addition, the Bonferroni correction was used to eliminate the false negatives of *p* values. SPSS (version 26) statistical software was utilized for statistical analyses, and the *p*‐value was considered .05 for statistical significance.

## 3 RESULTS

According to Table 1, vegan Turkish meatball recipes had significantly higher amounts of carbohydrates, dietary fiber, vitamin A, vitamin E, vitamin K, vitamin B1, vitamin B9, vitamin C, calcium, magnesium, ORAC, omega‐6, and glycemic index when compared to non‐vegan meatball recipes (*p* < .05). The total fat, cholesterol, protein, vitamin D, vitamin B2, vitamin B3, vitamin B12, zinc, and omega‐3 in Turkish vegan meatball recipes were significantly lower (*p* < .05). On the other hand, vegan English meatball recipes had higher amounts of carbohydrates, dietary fiber, vitamin B9, and magnesium when compared to non‐vegan versions (*p* < .05). In addition, cholesterol and vitamin B12 levels in English vegan meatball recipes were significantly lower than in non‐vegan meatball recipes (*p* < .05). Lastly, no statistically significant relationship was found between vegan and non‐vegan burger recipes (Table 2).

**TABLE 1:** Statistical analysis of vegan and non‐vegan meatball recipes.

	Turkish meatball recipes			English meatball recipes		
	Vegan (*n* = 41) median (IQR)	Non‐vegan (*n* = 41) median (IQR)	*p* value	Vegan (*n* = 12) median (IQR)	Non‐vegan (*n* = 12) median (IQR)	*p* value
Basic nutritional components in recipes						
Energy (kcal/100 g)	157.3 (127.2–235.9)	172.1 (161.3–187.4)	1.000	129.8 (88.1–220.65)	143 (107.55–194.25)	1.000
Carbohydrate (g/100 g)	18.9 (15.1–30.3)	3.8 (2.3–6.4)	<.001*	13.3 (7.4–25.6)	5.65 (3.25–6.65)	.022*
Total fat (g/100 g)	5.8 (1.5–6.5)	10.2 (9.5–11.3)	<.001*	4.3 (1.85–6.9)	8.65 (5.2–11.65)	1.000
Cholesterol (mg/100 g)	0 (0–0)	64 (44–73)	<.001*	0 (0–0)	50.45 (43.5–71.3)	<.001*
Protein (g/100 g)	7 (5.9–9.8)	15.2 (14–15.8)	<.001*	8.35 (4.8–16.65)	11.65 (8.4–14.15)	1.000
Dietary fiber (g/100 g)	5.4 (4.3–6.9)	0.6 (0.5–0.8)	<.001*	5 (2.8–6.6)	0.9 (0.55–1.15)	<.001*
Vitamin contents in recipes						
Vitamin A (μg/100 g)	76 (46–168)	37 (26–53)	<.001*	31 (13–47.85)	44.55 (29–74.8)	1.000
Vitamin D (μg/100 g)	0 (0–0)	0.1 (0–0.2)	<.001*	0.05 (0–0.4)	0.1 (0–0.3)	1.000
Vitamin E (mg/100 g)	1 (0.7–1.7)	0.4 (0.4–0.5)	<.001*	0.5 (0.25–0.85)	0.55 (0.35–0.7)	1.000
Vitamin K (μg/100 g)	26 (11–39)	0 (0–6)	<.001*	8.25 (2.65–18.35)	2.55 (0.55–5.1)	.659
Vitamin B1 (mg/100 g)	0.1 (0.1–0.2)	0.1 (0.1–0.1)	<.001*	0.1 (0.1–0.25)	0.1 (0.1–0.5)	1.000
Vitamin B2 (mg/100 g)	0.1 (0.1–0.1)	0.2 (0.2–0.2)	<.001*	0.1 (0.1–0.2)	0.1 (0.1–0.2)	1.000
Vitamin B3 (mg/100 g)	0.9 (0.6–1.5)	4.4 (4.1–4.9)	<.001*	1.4 (0.7–2.05)	2.65 (1.95–2.95)	.219
Vitamin B6 (mg/100 g)	0.2 (0.1–0.2)	0.2 (0.2–0.2)	1.000	0.1 (0.1–0.2)	0.15 (0.1–0.3)	1.000
Vitamin B9 (μg/100 g)	48 (40–76)	9 (8–10)	<.001*	31.4 (25.1–59.2)	12.7 (7.2–18.35)	.031*
Vitamin B12 (μg/100 g)	0 (0–0)	3 (2.7–3.2)	<.001*	0 (0–0)	0.5 (0.35–1.5)	<.001*
Vitamin C (mg/100 g)	10 (7–15.1)	3.4 (1.5–5)	<.001*	3.25 (1.6–4.85)	2.35 (1.15–11.3)	1.000
Mineral contents in recipes						
Sodium (mg/100 g)	367 (210–539)	359 (283–530)	1.000	193.95 (118.75–330.45)	217.4 (152.1–249.15)	1.000
Potassium (mg/100 g)	352 (285–440)	305 (293–322)	1.000	342.55 (262.75–619.35)	219.3 (187.7–272.4)	.339
Calcium (mg/100 g)	49 (39–63)	21 (17–24)	<.001*	36.35 (29.3–114.8)	36.9 (16–78.45)	1.000
Magnesium (mg/100 g)	47 (42–55)	22 (22–24)	<.001*	37.9 (32.55–92.95)	18.15 (15.55–26.3)	.014*
Phosphorus (mg/100 g)	129 (114–201)	159 (154–168)	.699	137.3 (89.7–239.5)	115.45 (93.7–137.75)	1.000
Iron (mg/100 g)	2 (1.9–2.6)	1.8 (1.7–2)	.193	1.5 (1.35–3.5)	1.35 (1.1–1.8)	1.000
Zinc (mg/100 g)	1.1 (0.9–1.4)	3.6 (3.3–3.8)	<.001*	0.85 (0.65–1.9)	2.1 (1.4–2.75)	.420
Other nutritional values						
ORAC	670 (329–896)	329 (231–382)	<.001*	360.75 (112.85–773.15)	189.35 (94.7–522.15)	1.000
Omega‐3	0.1 (0–0.1)	0.2 (0.1–0.2)	<.001*	0.25 (0.1–0.5)	0.1 (0.1–0.2)	1.000
Omega‐6	0.9 (0.5–2.6)	0.4 (0.3–0.4)	.003*	0.75 (0.4–2.35)	0.5 (0.3–1.05)	1.000
Glycemic index	6 (4–12)	0 (0–0)	<.001*	1.5 (0–16.35)	0.9 (0–2.45)	1.000

*Note*: Data present with median ± IQR.

* *p*‐value <.05.

**TABLE 2:** Statistical analysis of vegan and non‐vegan burger recipes.

	Turkish burger recipes			English burger recipes		
	Vegan (*n* = 6) median (IQR)	Non‐vegan (*n* = 6) median (IQR)	*p* value	Vegan (*n* = 6) median (IQR)	Non‐vegan (*n* = 6) median (IQR)	*p* value
Basic nutritional components in recipes						
Energy (kcal/100 g)	132.95 (96.2–210.6)	220.85 (188.3–254.3)	1.000	101.2 (68.5–208.4)	138.65 (125.3–226.2)	1.000
Carbohydrate (g/100 g)	17.4 (15.1–19.5)	22.6 (17.6–26.9)	1.000	17.65 (12.8–25.3)	9.55 (7.6–10.9)	1.000
Total fat (g/100 g)	5.45 (1.2–9.3)	9.5 (2.7–11)	1.000	1.75 (1–8)	8.05 (5.3–14.1)	1.000
Cholesterol (mg/100 g)	0 (0–0.1)	34.65 (29.6–57.6)	.061	0 (0–0)	50 (33.1–54.1)	.061
Protein (g/100 g)	5.05 (3.9–6.5)	12.1 (10.4–14.8)	.061	3.7 (3.1–7.5)	10.25 (4.6–15.5)	1.000
Dietary fiber (g/100 g)	2.9 (1.9–3.5)	1.05 (0.7–1.3)	.061	3.15 (1.9–3.3)	1.25 (0.8–1.6)	.364
Vitamin contents in recipes						
Vitamin A (μg/100 g)	57.15 (39.5–69.8)	25.95 (19.2–45.2)	1.000	47.3 (15.4–227.8)	40.55 (22–60.9)	1.000
Vitamin D (μg/100 g)	0 (0–0)	0.1 (0–0.2)	1.000	0 (0–0)	0.1 (0–0.3)	1.000
Vitamin E (mg/100 g)	0.6 (0.5–1.4)	1.3 (0.8–3.4)	1.000	0.9 (0.2–1.6)	0.65 (0.4–1.4)	1.000
Vitamin K (μg/100 g)	10.9 (3–15.9)	0.65 (0.3–1.3)	.242	9.8 (5.6–17.3)	3.4 (1.9–6.2)	1.000
Vitamin B1 (mg/100 g)	0.1 (0.1–0.2)	0.1 (0.1–0.1)	1.000	0.1 (0.1–0.2)	0.1 (0.1–0.2)	1.000
Vitamin B2 (mg/100 g)	0.05 (0–0.1)	0.2 (0.1–0.2)	1.000	0.1 (0–0.1)	0.2 (0.1–0.2)	.909
Vitamin B3 (mg/100 g)	0.7 (0.5–0.9)	3.1 (2.4–5.3)	.061	0.95 (0.5–1.4)	3.05 (1.9–4.1)	1.000
Vitamin B6 (mg/100 g)	0.1 (0.1–0.2)	0.1 (0.1–0.3)	1.000	0.2 (0.1–0.2)	0.2 (0.1–0.2)	1.000
Vitamin B9 (μg/100 g)	27.05 (20–32.4)	29.95 (12.9–31.8)	1.000	27.35 (14.6–47.3)	20.3 (13.3–35.9)	1.000
Vitamin B12 (μg/100 g)	0 (0–0)	1.45 (0.4–1.5)	.061	0 (0–0)	0.65 (0.3–2.5)	.061
Vitamin C (mg/100 g)	10.05 (7.8–21.8)	0.85 (0.4–1.2)	.061	9.35 (4.9–13.8)	4.7 (2.7–8)	1.000
Mineral contents in recipes						
Sodium (mg/100 g)	195.35 (109–443.8)	359.2 (241.2–604.4)	1.000	127.2 (68.8–226.9)	344.4 (204.2–574.6)	1.000
Potassium (mg/100 g)	199.45 (173.2–301.6)	219.5 (215–251.3)	1.000	232 (208.3–391.5)	261.25 (236.7–278.3)	1.000
Calcium (mg/100 g)	27.1 (15.4–44.7)	34.8 (26.4–48.7)	1.000	35.35 (24.6–44.5)	53.35 (25–80.7)	1.000
Magnesium (mg/100 g)	22.45 (12.7–54.2)	19.8 (18.8–21.1)	1.000	35 (23.3–38.5)	19.45 (19.1–22.9)	.727
Phosphorus (mg/100 g)	77 (36–143.1)	128.15 (121–153.2)	1.000	84.15 (62.6–134.2)	150.55 (99.2–185.3)	1.000
Iron (mg/100 g)	0.95 (0.5–1.8)	1.1 (1.1–1.2)	1.000	1.4 (1.2–1.7)	1.4 (0.9–1.6)	1.000
Zinc (mg/100 g)	0.5 (0.3–1.2)	1.85 (0.8–2.1)	1.000	0.65 (0.5–0.9)	1.25 (0.7–2.8)	1.000
Other nutritional values						
ORAC	292.2 (158.7–507.1)	117.85 (90.5–136.1)	1.000	413.25 (242.8–1046.7)	259.05 (110.9–313.4)	1.000
Omega‐3	0.05 (0–0.2)	0.1 (0–0.1)	1.000	0.1 (0–0.1)	0.1 (0–0.3)	1.000
Omega‐6	0.3 (0.2–1.5)	1.25 (0.8–3.1)	1.000	0.55 (0.3–1.2)	0.5 (0.3–2.1)	1.000
Glycemic index	1.7 (0–1.8)	19.25 (16–22.4)	.061	5.3 (2.8–9.9)	0.8 (0–3.2)	1.000

*Note*: Data present with median ± IQR.

The vegan Turkish pasta recipes had significantly higher amounts of dietary fiber and magnesium than non‐vegan pasta ingredients (Table 3; *p* < .05). The vegan English pasta recipes had statistically significantly higher dietary fiber and calcium (*p* < .05). The amounts of cholesterol, protein, vitamin B2, vitamin B3, vitamin B12, phosphorus, iron, and zinc in English vegan pasta recipes were significantly lower when compared to non‐vegan pasta recipes (*p* < .05).

**TABLE 3:** Statistical analysis of vegan and non‐vegan pasta recipes.

	Turkish pasta recipes			English pasta recipes		
	Vegan (*n* = 8) median (IQR)	Non‐vegan (*n* = 8) median (IQR)	*p* value	Vegan (*n* = 30) median (IQR)	Non‐vegan (*n* = 30) median (IQR)	*p* value
Basic nutritional components in recipes						
Energy (kcal/100 g)	271.75 (180.45–294.95)	195.15 (158.8–205)	1.000	129.55 (98.6–169.9)	159.1 (139–233)	.993
Carbohydrate (g/100 g)	32.4 (26.5–40.25)	22.45 (17.45–25.25)	1.000	15.45 (13–19.3)	14.85 (11.2–23.3)	1.000
Total fat (g/100 g)	8.3 (5.65–11.7)	6.15 (2–7.9)	1.000	3.75 (2.2–6.4)	6.5 (3.8–9.1)	1.000
Cholesterol (mg/100 g)	0 (0–0)	41 (21–56.5)	1.000	0 (0–0)	45.05 (29.4–56.6)	<.001*
Protein (g/100 g)	8.3 (5.95–9.15)	11.3 (7.85–13.7)	1.000	3.9 (3.2–5.7)	10.2 (7.9–12.7)	<.001*
Dietary fiber (g/100 g)	4.1 (3.45–4.9)	1.35 (0.95–2.05)	.013*	2.05 (1.6–2.6)	1.35 (1–2)	.020*
Vitamin contents in recipes						
Vitamin A (μg/100 g)	62.5 (9.5–115)	78 (60.5–88)	1.000	61.75 (32.3–124.9)	106.1 (75.9–189.6)	.269
Vitamin D (μg/100 g)	0 (0–0)	0 (0–0.1)	1.000	0 (0–0.3)	0.2 (0–0.3)	1.000
Vitamin E (mg/100 g)	1.55 (1.15–2.85)	0.65 (0.4–1.55)	1.000	0.5 (0.3–0.8)	0.6 (0.5–1.4)	1.000
Vitamin K (μg/100 g)	5 (2–13)	3.5 (1.8–10)	1.000	7.75 (4–32)	10 (3.1–34.1)	1.000
Vitamin B1 (mg/100 g)	0.1 (0.1–0.15)	0.1 (0.1–0.1)	1.000	0.1 (0–0.1)	0.1 (0.1–0.1)	.079
Vitamin B2 (mg/100 g)	0.1 (0–0.1)	0.1 (0.1–0.1)	1.000	0.1 (0–0.1)	0.1 (0.1–0.1)	<.001*
Vitamin B3 (mg/100 g)	1.2 (1.05–1.35)	3.25 (1.4–5)	.992	0.6 (0.5–0.9)	2 (1.4–3)	<.001*
Vitamin B6 (mg/100 g)	0.2 (0.1–0.2)	0.2 (0.1–0.3)	1.000	0.1 (0.1–0.1)	0.1 (0.1–0.2)	1.000
Vitamin B9 (μg/100 g)	30 (25–41.5)	18 (12.5–23)	.065	20.9 (15.2–32.1)	19.05 (15.8–27.3)	1.000
Vitamin B12 (μg/100 g)	0 (0–0)	0.2 (0.05–0.6)	.352	0 (0–0)	0.2 (0.1–0.6)	<.001*
Vitamin C (mg/100 g)	3.1 (0.7–20.45)	8.8 (3.05–13.5)	1.000	8.65 (5.5–11.5)	8.65 (4.8–15.6)	1.000
Mineral contents in recipes						
Sodium (mg/100 g)	280.5 (153–446)	185 (131.5–304.1)	1.000	133.45 (55.3–191.4)	175.95 (69.6–305.3)	1.000
Potassium (mg/100 g)	273.5 (216.5–313)	238.45 (223.5–249.5)	1.000	185.05 (127.7–220.9)	220.65 (161.8–292)	.354
Calcium (mg/100 g)	29 (25–43)	23 (19–30)	1.000	27.5 (21.3–47.3)	81.3 (37.8–123.6)	.001*
Magnesium (mg/100 g)	46 (37–62.5)	28 (21–28)	.013*	24.4 (18.2–29.4)	27.15 (20.7–35.6)	1.000
Phosphorus (mg/100 g)	124.5 (100–154.5)	124 (71.3–149.5)	1.000	65.2 (48.9–78.8)	136.5 (106.1–159.6)	<.001*
Iron (mg/100 g)	1.45 (1.1–2.45)	1.5 (1.15–1.6)	1.000	0.9 (0.6–1.1)	1.5 (1.1–2)	.001*
Zinc (mg/100 g)	0.95 (0.8–1.4)	0.85 (0.65–1.15)	1.000	0.55 (0.4–0.7)	1.1 (0.9–1.4)	<.001*
Other nutritional values						
ORAC	340 (225.5–1151.5)	261 (180.95–310.5)	1.000	369 (250.3–630.3)	227.4 (152–786.8)	1.000
Omega‐3	0.1 (0–0.15)	0.1 (0.05–0.1)	1.000	0 (0–0.1)	0.1 (0.1–0.1)	.191
Omega‐6	1.9 (0.85–3.3)	0.6 (0.4–1.3)	1.000	0.45 (0.3–0.9)	0.5 (0.3–0.8)	1.000
Glycemic index	3 (0.5–10.5)	8 (0–10)	1.000	0.6 (0–2)	6.15 (0–12.5)	.166

*Note*: Data present with median ± IQR.

* *p*‐value <.05.

According to Table 4, non‐vegan English pizza recipes had significantly higher cholesterol and vitamin B9 values than non‐vegan versions (*p* < .05). Among the cake recipes posted as Turkish, vegan recipes had significantly lower amounts of energy, total fat, cholesterol, protein, vitamin B2, vitamin B12, and omega‐6 compared to non‐vegan cake recipes (*p* < .05). The dietary fiber and vitamin B3 in Turkish vegan cake recipes were significantly higher than in non‐vegan cake recipes (*p* < .05). Similarly, the non‐vegan English cake recipes had higher cholesterol, protein, vitamin B2, and vitamin B12 levels than vegan cake recipes (*p* < .05) (Table 5).

**TABLE 4:** Statistical analysis of vegan and non‐vegan pizza recipes.

	Turkish pizza recipes			English pizza recipes		
	Vegan (*n* = 6) median (IQR)	Non‐vegan (*n* = 6) median (IQR)	*p* value	Vegan (*n* = 9) median (IQR)	Non‐vegan (*n* = 9) median (IQR)	*p* value
Basic nutritional components in recipes						
Energy (kcal/100 g)	164.2 (143.1–189.2)	226.2 (211.3–285.7)	.061	125.7 (117.2–215.1)	159.9 (130.5–216.3)	1.000
Carbohydrate (g/100 g)	18.6 (14.6–26.3)	27.8 (24.7–39.2)	1.000	19.5 (11.5–29.3)	17.7 (12.9–30.2)	1.000
Total fat (g/100 g)	7 (4.5–8.6)	9.2 (7.8–10.2)	1.000	6 (2.7–7.7)	7.1 (6.5–9.5)	1.000
Cholesterol (mg/100 g)	0.05 (0–5.5)	13.65 (11.7–18)	.424	0 (0–0)	10 (9–17)	.001*
Protein (g/100 g)	4.5 (4.2–6.8)	7.5 (6.6–8.8)	1.000	5 (3.8–6.2)	6.1 (5.1–8.7)	1.000
Dietary fiber (g/100 g)	2.1 (1.6–4.7)	1.9 (1.7–2.1)	1.000	1.7 (1.6–2.9)	1.7 (1.4–2)	1.000
Vitamin contents in recipes						
Vitamin A (μg/100 g)	97.75 (54.5–117.6)	77.65 (74.4–103.1)	1.000	34.8 (23.6–70.6)	87 (71–164)	.525
Vitamin D (μg/100 g)	0.05 (0–1)	0.2 (0.1–0.3)	1.000	0 (0–0.1)	0.1 (0–0.2)	1.000
Vitamin E (mg/100 g)	1.05 (0.8–1.1)	1.5 (1–2.2)	1.000	0.5 (0.4–0.7)	1 (0.6–1.9)	1.000
Vitamin K (μg/100 g)	9.25 (6–18.3)	3.8 (2–4.9)	1.000	14.7 (8.2–20.2)	5 (4–13)	1.000
Vitamin B1 (mg/100 g)	0.1 (0.1–0.2)	0.1 (0.1–0.1)	1.000	0.1 (0.1–0.1)	0.1 (0–0.1)	1.000
Vitamin B2 (mg/100 g)	0.1 (0.1–0.1)	0.15 (0.1–0.2)	1.000	0.1 (0.1–0.1)	0.1 (0.1–0.1)	1.000
Vitamin B3 (mg/100 g)	0.75 (0.7–1.8)	1.2 (1.2–1.2)	1.000	0.9 (0.7–1.3)	1.1 (0.7–1.3)	1.000
Vitamin B6 (mg/100 g)	0.1 (0.1–0.2)	0.1 (0.1–0.1)	1.000	0.1 (0.1–0.1)	0.1 (0.1–0.1)	1.000
Vitamin B9 (μg/100 g)	46.25 (29.7–57.2)	34.05 (28.3–41)	1.000	1 (0–1)	27 (17–34)	.001*
Vitamin B12 (μg/100 g)	0 (0–0.1)	0.3 (0.3–0.4)	.303	0.1 (0–0.3)	0.2 (0.2–0.2)	1.000
Vitamin C (mg/100 g)	8.9 (4.7–21.2)	10.4 (5.5–18.2)	1.000	9.9 (3.7–15.2)	7.1 (5.9–11.3)	1.000
Mineral contents in recipes						
Sodium (mg/100 g)	281.3 (246.8–469.7)	344.5 (234.9–402.2)	1.000	298.4 (186.8–324.7)	278 (200–348)	1.000
Potassium (mg/100 g)	246.25 (182.2–324.6)	196.45 (181–214)	1.000	270 (233–296.7)	186 (183–233)	1.000
Calcium (mg/100 g)	37.65 (30.2–54.9)	76.35 (66–94.5)	1.000	26.2 (18.3–40.8)	70 (51–85)	.397
Magnesium (mg/100 g)	20.65 (13.5–46.8)	21.15 (16.6–21.3)	1.000	25.5 (16.2–46.2)	17 (12–22)	1.000
Phosphorus (mg/100 g)	69.65 (51.9–134.5)	108.2 (91.6–118.5)	1.000	45.1 (43.3–120.2)	114 (83–124)	1.000
Iron (mg/100 g)	1.05 (0.6–1.4)	0.65 (0.6–0.8)	1.000	1.3 (0.7–1.7)	0.8 (0.6–1.2)	1.000
Zinc (mg/100 g)	0.5 (0.4–1)	0.85 (0.7–0.9)	1.000	0.4 (0.3–0.9)	0.6 (0.5–0.8)	1.000
Other nutritional values						
ORAC	393.05 (275.2–533.9)	170.4 (150.6–262)	1.000	257.7 (199.5–541)	488 (173–1059)	1.000
Omega‐3	0.1 (0.1–0.2)	0.1 (0.1–0.1)	1.000	0 (0–0.1)	0.1 (0.1–0.1)	1.000
Omega‐6	0.6 (0.5–0.8)	1.15 (0.6–1.9)	1.000	0.4 (0.2–1.4)	0.6 (0.4–0.7)	1.000
Glycemic index	11.7 (5.9–17.2)	22.45 (17.9–30.8)	1.000	12.8 (0–18.1)	3 (0–9)	1.000

*Note*: Data present with median ± IQR.

* *p*‐value <.05.

**TABLE 5:** Statistical analysis of vegan and non‐vegan cake recipes.

	Turkish cake recipes			English cake recipes		
	Vegan (*n* = 34) median (IQR)	Non‐vegan (*n* = 34) median (IQR)	*p* value	Vegan (*n* = 24) median (IQR)	Non‐vegan (*n* = 24) median (IQR)	*p* value
Basic nutritional components in recipes						
Energy (kcal/100 g)	267.7 (234.1–298.5)	320.55 (297.6–342.7)	.007*	272.3 (212.15–323.65)	316.75 (291.95–356.95)	.155
Carbohydrate (g/100 g)	39.05 (31.6–44.2)	37 (33.9–41.1)	1.000	39.9 (32.85–46.2)	39.7 (29.9–43.3)	1.000
Total fat (g/100 g)	9.25 (7.4–13.7)	16.45 (12.5–18.6)	.004*	11.4 (4.95–13.45)	17.3 (11.25–20.45)	.242
Cholesterol (mg/100 g)	0 (0–0)	78.5 (66–90)	<.001*	0 (0–0)	72.8 (31.75–104.35)	<.001*
Protein (g/100 g)	3.45 (3–4.4)	5.7 (5–6.3)	<.001*	4.2 (3.8–4.8)	5.6 (4.6–7.1)	.008*
Dietary fiber (g/100 g)	2 (1.4–3.1)	1 (0.8–1.3)	<.001*	2.2 (1.55–3)	1.4 (1–2.4)	.921
Vitamin contents in recipes						
Vitamin A (μg/100 g)	6 (1–144)	38 (32–57)	1.000	13.1 (1.85–129.45)	79.85 (20.7–174.9)	.478
Vitamin D (μg/100 g)	0 (0–15.2)	0.4 (0.3–0.5)	.352	0.1 (0–13)	0.4 (0.2–0.5)	1.000
Vitamin E (mg/100 g)	4.15 (1.4–5.3)	6.25 (4–9.1)	.146	1.8 (0.4–7.4)	3.15 (0.6–6.35)	1.000
Vitamin K (μg/100 g)	1 (1–2)	1 (1–1)	1.000	0.8 (0.4–1.7)	1.25 (0.85–2.1)	1.000
Vitamin B1 (mg/100 g)	0.1 (0–0.1)	0.1 (0.1–0.1)	1.000	0.1 (0–0.1)	0.1 (0–0.1)	1.000
Vitamin B2 (mg/100 g)	0 (0–0.1)	0.1 (0.1–0.1)	<.001*	0 (0–0.1)	0.1 (0.1–0.1)	.002*
Vitamin B3 (mg/100 g)	0.4 (0.2–0.7)	0.2 (0.2–0.3)	.008*	0.4 (0.3–0.6)	0.3 (0.3–0.6)	1.000
Vitamin B6 (mg/100 g)	0.1 (0–0.1)	0 (0–0.1)	.648	0.1 (0–0.1)	0.05 (0–0.1)	1.000
Vitamin B9 (μg/100 g)	9.5 (4–18)	14 (13–17)	.780	13.05 (7.15–20)	13.35 (10.35–17.55)	1.000
Vitamin B12 (μg/100 g)	0 (0–0)	0.2 (0.2–0.3)	<.001*	0 (0–0.1)	0.2 (0.1–0.3)	<.001*
Vitamin C (mg/100 g)	1.9 (0–3.9)	0.5 (0.3–3.1)	1.000	3.55 (2.35–5.2)	0.45 (0.3–5.55)	.663
Mineral contents in recipes						
Sodium (mg/100 g)	166 (115–216)	150 (129–188)	1.000	136.8 (57.85–204.05)	123.35 (64.6–272.7)	1.000
Potassium (mg/100 g)	140.5 (105–282)	111.5 (106–156)	1.000	166.6 (89.6–268.75)	159.65 (117.7–286.45)	1.000
Calcium (mg/100 g)	41.5 (22–89)	50.5 (44–63)	1.000	50.3 (29.7–99.95)	40.45 (36.85–52.5)	1.000
Magnesium (mg/100 g)	18.25 (12–48)	13.5 (9–19)	.182	22.95 (11.15–44.95)	19.85 (10.4–36.9)	1.000
Phosphorus (mg/100 g)	129 (100–171)	163.5 (138–180)	.530	126.75 (87.9–174.9)	110.15 (76.9–131.7)	1.000
Iron (mg/100 g)	0.9 (0.6–1.9)	0.7 (0.6–1.1)	1.000	0.7 (0.45–1.3)	1.15 (0.6–2.4)	1.000
Zinc (mg/100 g)	0.4 (0.2–0.9)	0.5 (0.5–0.7)	1.000	0.5 (0.25–0.8)	0.5 (0.4–0.75)	1.000
Other nutritional values						
ORAC	926.5 (270.4–1736)	1241.5 (964–1544)	1.000	176.3 (47.3–446.1)	886.95 (32.7–1680.9)	1.000
Omega‐3	0 (0–0.1)	0.1 (0–0.1)	1.000	0.05 (0–0.1)	0.1 (0–0.1)	1.000
Omega‐6	3.8 (2.5–5)	6.35 (4.7–8.2)	.035*	2.35 (0.85–6.65)	3.4 (0.7–6.1)	1.000
Glycemic index	16.5 (12–27)	15.85 (11–18)	1.000	20.25 (10.55–27.45)	16.95 (12.7–21.15)	1.000

*Note*: Data present with median ± IQR.

* *p*‐value <.05.

Vegan Turkish cookie recipes had significantly higher amounts of dietary fiber, vitamin B1, vitamin B3, vitamin B6, vitamin B9, potassium, calcium, magnesium, phosphorus, iron, and zinc when compared to non‐vegan cookie recipes (*p* < .05). The total fat, cholesterol, vitamin A, vitamin D, and vitamin B12 levels in Turkish vegan cookie recipes were significantly lower than in non‐vegan recipes (*p* < .05). In addition, vegan English cookie recipes had significantly lower amounts of cholesterol, vitamin A, vitamin B2, vitamin B12, and omega‐6 than non‐vegan cookie recipes (*p* < .05) (Table 6).

**TABLE 6:** Statistical analysis of vegan and non‐vegan cookie recipes.

	Turkish cookie recipes			English cookie recipes		
	Vegan (*n* = 31) median (IQR)	Non‐vegan (*n* = 31) median (IQR)	*p* value	Vegan (*n* = 21) median (IQR)	Non‐vegan (*n* = 21) median (IQR)	*p* value
Basic nutritional components in recipes						
Energy (kcal/100 g)	408 (300–448.9)	461.5 (416.1–477.3)	.074	411.8 (358.1–435.2)	427.3 (404.5–461)	1.000
Carbohydrate (g/100 g)	46.9 (36.7–55.6)	50.9 (44.5–55.1)	1.000	53.2 (37.3–57.2)	49.3 (44.6–52.7)	1.000
Total fat (g/100 g)	18.5 (11.3–23.4)	23.6 (20–30.2)	.037*	16.3 (14.2–24.4)	20.9 (18.6–27.7)	1.000
Cholesterol (mg/100 g)	0 (0–0)	57 (35–80)	<.001*	0 (0–0)	79 (68–96.7)	<.001*
Protein (g/100 g)	6.5 (5.2–10.8)	5.8 (4.8–7.4)	1.000	5.4 (4.2–6.8)	6.7 (5.1–10)	1.000
Dietary fiber (g/100 g)	4 (2.7–5.3)	1.5 (1.2–2.2)	<.001*	2 (1.1–3.4)	1.4 (1.2–3.2)	1.000
Vitamin contents in recipes						
Vitamin A (μg/100 g)	1 (1–13)	135.8 (111–164)	<.001*	0.4 (0–21.7)	140.2 (91.6–166.1)	<.001*
Vitamin D (μg/100 g)	0 (0–0)	0.4 (0.3–0.6)	<.001*	0 (0–0.1)	0.4 (0.4–0.5)	.156
Vitamin E (mg/100 g)	2.2 (0.5–5.3)	3.9 (1.5–5.1)	1.000	0.2 (0.1–1.5)	0.7 (0.6–1.7)	.123
Vitamin K (μg/100 g)	2 (1–7)	2 (2–5)	1.000	0.7 (0–6)	1.7 (1.4–2.4)	1.000
Vitamin B1 (mg/100 g)	0.2 (0.1–0.3)	0.1 (0–0.1)	<.001*	0.1 (0–0.1)	0.1 (0.1–0.1)	1.000
Vitamin B2 (mg/100 g)	0.1 (0–0.1)	0.1 (0–0.1)	1.000	0 (0–0)	0.1 (0.1–0.2)	<.001*
Vitamin B3 (mg/100 g)	0.8 (0.5–1.4)	0.3 (0.3–0.4)	.001*	0.4 (0.3–0.5)	0.3 (0.3–0.7)	1.000
Vitamin B6 (mg/100 g)	0.1 (0.1–0.3)	0 (0–0.1)	<.001*	0 (0–0.1)	0 (0–0.1)	1.000
Vitamin B9 (μg/100 g)	26 (9–34)	9 (7–12)	.028*	9.4 (4.6–19)	15.3 (8.3–28)	1.000
Vitamin B12 (μg/100 g)	0 (0–0)	0.1 (0–0.1)	<.001*	0 (0–0.1)	0.1 (0.1–0.2)	.002*
Vitamin C (mg/100 g)	0.4 (0–5.7)	0.1 (0–0.3)	1.000	0.1 (0–3.7)	0.1 (0.1–0.2)	1.000
Mineral contents in recipes						
Sodium (mg/100 g)	209 (21–304.7)	128.1 (83–186)	1.000	114.3 (82.5–208)	232.7 (93.4–322.8)	1.000
Potassium (mg/100 g)	318 (107–440)	120 (93–171)	.013*	215.4 (102.6–326.5)	160.1 (95–271.8)	1.000
Calcium (mg/100 g)	56 (31–132)	26.7 (17–45)	.007*	34.3 (22.6–45)	43.4 (25.8–76.7)	1.000
Magnesium (mg/100 g)	58 (20–69)	13 (9–23)	.008*	32.8 (12.1–63.6)	23.7 (13.3–70.8)	1.000
Phosphorus (mg/100 g)	205 (139–315)	146 (95–156)	.019*	77.2 (53–125.7)	143.6 (87–222.6)	.080
Iron (mg/100 g)	1.9 (1–2.9)	0.7 (0.5–1.1)	.004*	0.9 (0.4–2.9)	0.9 (0.5–1.7)	1.000
Zinc (mg/100 g)	1.1 (0.6–1.8)	0.4 (0.4–0.7)	.004*	0.7 (0.2–1.1)	0.7 (0.5–1.5)	1.000
Other nutritional values						
ORAC	1284 (5–2557)	1219 (31–1762)	1.000	391.3 (34.3–614)	501 (0–1420.3)	1.000
Omega‐3	0.1 (0–0.4)	0.1 (0.1–0.6)	1.000	0 (0–0.3)	0.1 (0.1–0.2)	1.000
Omega‐6	3.8 (1.9–6.3)	4.2 (2.5–8.1)	1.000	0.3 (0.2–0.9)	1.2 (0.7–3)	.041*
Glycemic index	17.1 (0–29)	23.6 (20–26)	1.000	18.7 (11.4–23.9)	14.5 (9.2–20.8)	1.000

*Note*: Data present with median ± IQR.

* *p*‐value <.05.

## 4 DISCUSSION

The current study investigated the nutritional composition of vegan recipes on Instagram. To the best of our knowledge, this study has unique value, being different from other studies in the literature due to the application of data science with social media in vegan nutrition. This innovative approach identifies nutrient profiles in food recipes. In this way, alternative plant‐based ingredients can be added to poorly planned vegan recipes, and more nutrients in vegan recipes can provide sustainable well‐being.

According to our findings, Turkish recipes of cakes had significantly lower energy than non‐vegan versions (*p* < .05). The level of carbohydrates was significantly higher in vegan meatball recipes (*p* < .05). However, the total fat levels of vegan recipes, including meatballs posted in English with meatballs, cake, and cookies shared in Turkish, were significantly lower when compared to their non‐vegan versions (*p* < .05). In the literature, several studies focused on the nutritional content of commercial vegan products. Although these studies did not include the same food groups as the current study, excessive amounts of carbohydrates with a deficiency in energy and fat in vegan foods drew attention (Alexy et al., 2021; Schüpbach et al., 2017; Turner‐McGrievy et al., 2015). In this context, vegan substitute products (*n* = 152) in the Brazilian market were investigated by Romão et al. (2022). Only vegan versions of cheeses and beverages had higher levels of carbohydrates and dietary fiber when compared to their non‐vegan versions (Romão et al., 2022). Plant‐based meat and milk analogs (*n* = 421) were compared with animal products in another study. According to Katidi et al. (2023), artificial sausages included more protein and less salt, total fat, and saturated fat than their animal‐based counterparts. Plant‐based milk and yogurt analogs had lower levels of protein but higher levels of carbohydrates when compared to their animal‐based counterparts (Katidi et al., 2023).

One of the most common problems in a poorly planned vegan diet is also the lack of essential amino acids since the limie of most fruits, vegetables, and cereals are limited (Hovinen et al., 2021; Özcan & Baysal, 2016). However, this requirement could be easily met by providing a variety of plant‐based foods and consuming a balanced diet of essential amino acids. The current study provided the amount of protein in recipes instead of the essential amino acids due to the lack of a menu analysis. As expected, the Turkish vegan recipes of meatballs and cake had significantly lower amounts of protein. In contrast, the English vegan pasta and cake recipes had significantly lower protein levels (*p* < .05). Guess et al. (2023) compared to the 1507 meat‐containing items, with 81 vegan and 191 vegetarian choices on restaurant menus. Similar to current data, the vegetarian and vegan recipes had significantly lower protein contents when compared to dishes that contain meat (*p* < .001) (Guess et al., 2023). In another study, a few of 295 plant‐based cheeses in the United States could be considered good dietary protein sources. Therefore, non‐dairy cheese alternatives should not be viewed as a healthy alternative to dairy cheese (Craig et al., 2022). In addition to the low protein levels in vegan recipes, the bioavailability of the amino acids was also critical. In the study of Sousa et al. (2023), the protein content of two extensively processed vegetable burgers, derived from either soy or pea‐faba proteins, was contrasted with that of beef burgers. In this context, the grilled beef burger exhibited the highest in vitro digestible amino acid ratio (DIAAR) values (Sousa et al., 2023). In vitro, the digestibility of amino acids in different recipes could be studied.

In this study, the dietary fiber levels in meatball and pasta recipes were significantly higher than in their non‐vegan versions (*p* < .05). In addition, Turkish cake and cookie recipes had significantly higher dietary fiber levels than non‐vegan versions (*p* < .05). Consistently, the high dietary fiber level in vegan products and the high dietary fiber consumption of vegans have been noted in many studies (Katidi et al., 2023; Raman et al., 2023; Romão et al., 2022; Seel et al., 2023). High dietary fiber intake has many health benefits, such as a lower risk of developing obesity, diabetes, coronary heart disease, stroke, hypertension, and certain gastrointestinal diseases (Anderson et al., 2009). Nevertheless, high dietary fiber intake also affects gut microbiota. In connection with the situation, Raman et al. (2023) have demonstrated that greater consumption of dietary fiber has contributed to a decrease in the diversity of the microbiome profile in vegans compared to omnivores (Raman et al., 2023). Similarly, a non‐randomized, controlled, and prospective study with 2288 participants confirmed their findings (Seel et al., 2023).

Vegan recipes are cholesterol‐free since cholesterol is a structural component of animal cell membranes (Lütjohann et al., 2018). Although vegans have lower total and LDL cholesterol levels than omnivores, it is challenging to achieve reduced levels of cardiovascular risk factors like blood pressure and BMI to maintain the favorable outcomes associated with a vegan lifestyle (Lütjohann et al., 2018; Weikert et al., 2020). It entails incorporating a broader range of food sources within a specific dietary framework (Raman et al., 2023).

Despite practically comparable macronutrient levels, discrepancies emerged between vegan and non‐vegan recipes in micronutrient levels. In particular, between vegan and non‐vegan recipes, there were inconsistent findings in the vitamin A, vitamin E, vitamin K, vitamin B1, vitamin B3, vitamin B9, potassium, calcium, phosphorus, iron, and zinc levels. Compared with the literature, differences stood out between the nutritional status of vegans in the current study. For instance, Weikert et al. (2020) demonstrated that vegans consumed far more dietary fiber, vitamin E, vitamin K, folate, and iron than omnivores (Weikert et al., 2020). However, English pasta and pizza recipes had lower iron levels (*p* < .05). At this point, our findings were only related to analyzing the food composition of recipes. Utilization of different alternative ingredients and plant‐based dietary supplements could contribute to understanding these contradictions.

The current paper showed that the vitamin D levels in Turkish vegan meatball and cookie recipes were significantly lower than in non‐vegan versions (*p* < .05). Consistently, several studies reported that vegans had lower vitamin D intake compared to omnivores (Davey et al., 2003; Weikert et al., 2020). These findings suggested lower vitamin D concentrations appeared in poorly planned vegan diets. On the other hand, there were no significant differences in 25‐OH vitamin D3 levels between modern vegetarian, vegan, and omnivorous children and adolescents in the Vechi Youth Study (Alexy et al., 2021). Moreover, a high prevalence (>30%) of 25‐OH vitamin D3 deficiency was determined irrespective of the diet group. These outcomes indicated that vitamin D deficiency could not be a specific nutritional risk for vegans and vegetarians. It is a public health concern that causes many complications, such as bone diseases and diabetes, and must be treated throughout society (Gökcen et al., 2019; Holick et al., 2011).

In the current study, nearly all of the vegan recipes had significantly lower B12 vitamin levels when compared to their non‐vegan versions (*p* < .05). Similarly, Harnack et al. demonstrated that 37 plant‐based ground beef substitutes (*n* = 37) in the United States markets were deficient in vitamin B12 compared to ground beef (Harnack et al., 2021). Consistently, according to Weikert et al. (2020), the vitamin B12 status of vegans was similar to that of non‐vegans, thanks to the B12 vitamin supplementation. The study by Weikert et al. included a particular German vegan population with unique sociodemographic characteristics and was restricted to the Berlin area in Germany. Therefore, the results could not be generalized to the vegan population (Weikert et al., 2020). Nevertheless, Storz et al. supported that supplementing vegans with vitamin B12 provided a sufficient B12 level comparable to omnivores (Storz et al., 2023). In addition, Selinger et al. reported that vitamin B12 deficiency was prevalent among Czech vegans who did not utilize B12 supplements (Selinger et al., 2019). Consuming a balanced diet, including supplements or fortified products, could be sufficient to prevent micronutrient intake deficiencies in vegans (Schüpbach et al., 2017).

The antioxidant status in vegans was much higher than that of omnivores due to the inclusion of antioxidant‐rich foods (Szeto et al., 2004; Trapp et al., 2010). Interestingly, ORAC values were greater only in vegan meatball recipes posted as Turkish in the current study. As in our findings, Haldar et al. (2007) demonstrated no significant differences in antioxidant status between vegetarians and omnivores (Haldar et al., 2007). Vanacore et al. (2018) confirmed that a restrictive vegan diet could not protect against the development of metabolic and cardiovascular diseases (Vanacore et al., 2018). At this point, the antioxidant capacity of plant‐based recipes may be exaggerated. Lastly, plant‐based vegan diets may be recommended to prevent and treat type 2 diabetes because of their lower GI levels (McMacken & Shah, 2017). However, the glycemic index (GI) value was higher in Turkish vegan meatball recipes than in non‐vegan versions. It could be related to the ingredients of the recipes.

Lastly, omega‐3 levels in Turkish vegan meatball recipes were lower in non‐vegan versions (*p* < .05). Welch et al. reported that intake of total dietary omega‐3 fatty acids (g/d), ALA (g/d), EPA (g/d), and DHA (g/d) were lower in vegans than meat‐eaters, fish‐eaters, and vegetarians. Besides that, Burns‐Whitmore et al. showed that not all vegan diets met the Dietary Reference Intake (DRI) adequate intake for dietary ALA. In addition, vegan diets had high intakes of linoleic acid (LA) as compared to omnivore/non‐vegetarian diets. It should be considered that high intakes of LA competitively interfere with the endogenous conversion of ALA to EPA and DHA (Burns‐Whitmore et al., 2019).

This study had several strengths in presenting a wide range of data about recipes in different languages and food groups, such as meatballs, burgers, pasta, pizza, cake, and cookies. The limitation of the study was that it included limited vegan recipes shared for one year only on Instagram.

## 5 CONCLUSION

The evaluated vegan recipes had higher dietary fiber content but lower B12 vitamin and cholesterol content than non‐vegan versions. The most significant differences in nutritional composition were determined between vegan and non‐vegan meatball recipes. Additionally, differences between culinary cultures have been reflected in vegan recipes. Nevertheless, it would not be correct to associate the nutritional deficiencies in vegan individuals with a single recipe here. Consuming different vegan foods throughout the day could provide complementary nutrient intake and sustainable optimal health. In addition, recipe‐based revisions could be an innovative approach to improve the nutrition status of vegans in the future. More comprehensive research in different languages and social media tools could contribute to the quality of life among vegans. In addition, analysis of vegan recipes could be utilized to modulate vegan supplementation programs.

## AUTHOR CONTRIBUTIONS

**Tuba Yoldaş:** Conceptualization (equal); investigation (equal); methodology (equal); resources (equal). **Gözde Sultan Kaya:** Conceptualization (equal); investigation (equal); methodology (equal); resources (equal). **Ayhan Parmaksız:** Methodology (lead). **Handan Işıklar:** Investigation (equal); writing – review and editing (equal). **Elif Günalan:** Conceptualization (equal); investigation (equal); methodology (equal); resources (equal); supervision (equal); writing – review and editing (equal).

## CONFLICT OF INTEREST STATEMENT

None.

## ETHICS STATEMENT

This study does not require ethical approval including open‐access food analysis.

## Supporting information


Figure S1.

## Data Availability

We can share all the study data.
