# Supplementary figures and images for: Nutritional analysis of vegan recipes: From social media to plate
Source: Food Sci Nutr. 2024 Aug 2;12(10):7657–70. doi: 10.1002/fsn3.4382 (PMC11521697; doi:10.1002/fsn3.4382)

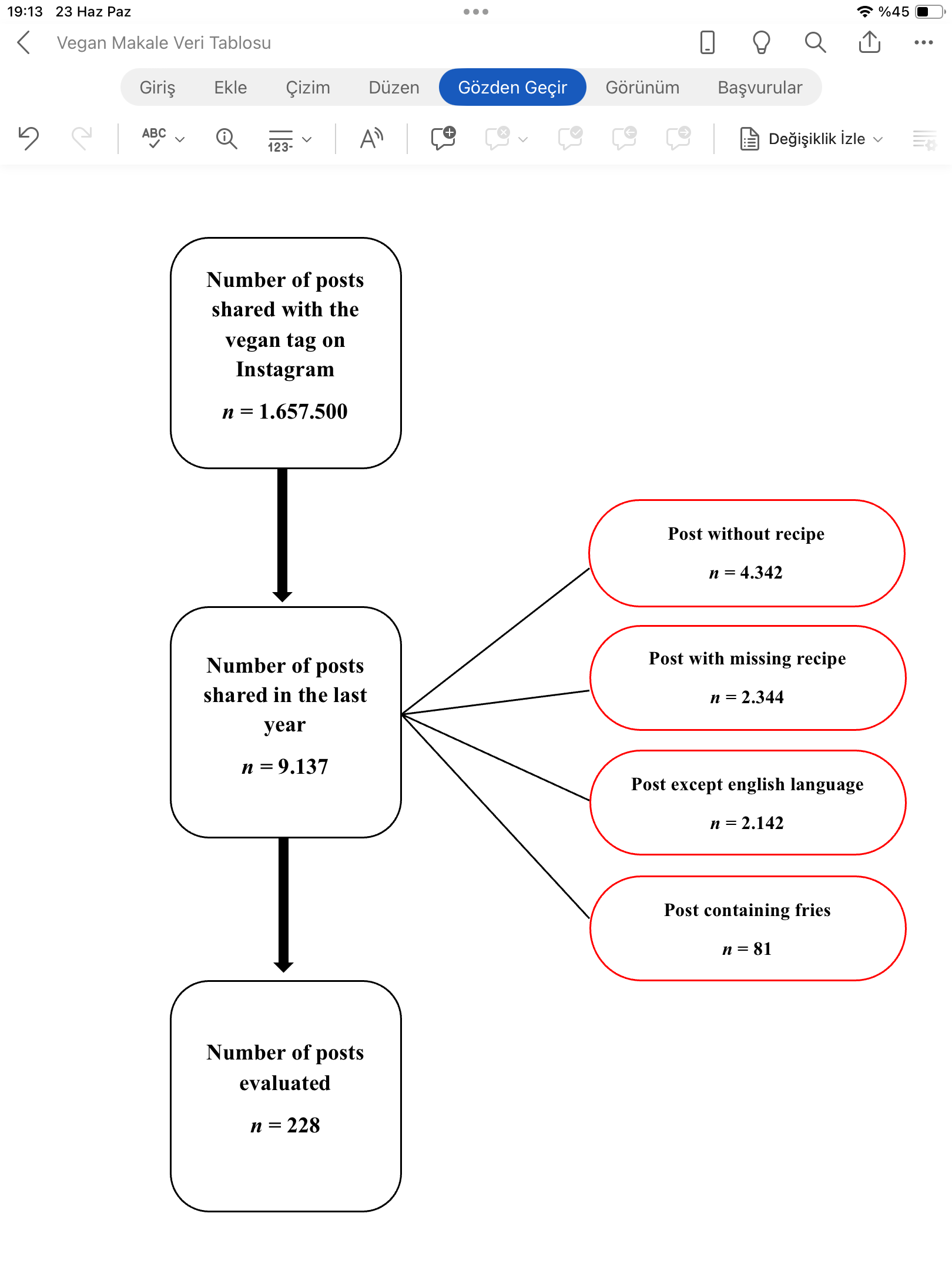


Figure S1. Flowchart of inclusion and exclusion of vegan recipes

Supplement: Supplementary file 1 — Figure S1. [file FSN3-12-7657-s001.docx]
